# Global brain network dynamics predict therapeutic responsiveness to cannabidiol treatment for refractory epilepsy

**DOI:** 10.1093/braincomms/fcaa140

**Published:** 2020-08-31

**Authors:** David E Anderson, Deepak Madhavan, Arun Swaminathan

**Affiliations:** Department of Ophthalmology & Visual Sciences, University of Nebraska Medical Center, Omaha, NE 68198-5540, USA; Department of Neurological Sciences, University of Nebraska Medical Center, Boys Town, NE 68010, USA; Department of Pediatric Neurology, Boys Town National Research Hospital, Omaha, NE 68198-8440, USA; Department of Neurological Sciences, University of Nebraska Medical Center, Boys Town, NE 68010, USA

**Keywords:** refractory epilepsy, EEG, graph theory, cannabidiol, anti-epileptic drugs

## Abstract

Refractory epilepsy is a chronic brain network disorder characterized by unresponsiveness to multiple (>2) anti-epileptic drugs. Cannabidiol, a non-psychotropic neuroactive substance, is an emerging anti-epileptic treatment that was recently approved by the US Food and Drug Administration for the treatment of refractory epilepsy, especially Lennox Gastaut syndrome and Dravet syndrome. Here, we evaluated associations between global brain network dynamics and related changes and responsiveness to cannabidiol therapy using a combination of electroencephalography phase coherence and graph theoretical analyses. Refractory epilepsy patients with Lennox Gastaut syndrome or Dravet syndrome underwent serial electroencephalography testing prior to and during cannabidiol treatment. Patients showing greater than 70% seizure frequency reduction were classified as treatment responders for the purposes of this study. We calculated inter-electrode electroencephalography phase coherence in delta (1–3 Hz), theta (4–7 Hz), alpha (8–12 Hz) and beta (13–30 Hz) frequency bands. Graph theoretical analysis of brain network dynamics was extracted from phase coherence to evaluate measures of network integration (i.e. characteristic path length, global efficiency and degree) and segregation (i.e. modularity and transitivity). We found that responders, relative to non-responders, showed increased network integration—as indexed by relatively higher global efficiency and lower degree—and increased network segregation—as indexed by relatively higher modularity—exclusively in the beta-frequency band. We also found that larger cannabidiol dosages were associated with increased network integration—as indexed by higher global efficiency with increasing dose—and increased network segregation—as indexed by lower transitivity with increasing dose—in the delta, theta and alpha frequency bands. In summary, we demonstrate novel effects of cannabidiol on brain network dynamics with important implications for the treatment of refractory epilepsy and, possibly, across broader research applications in the future.

## Teaser

Cannabidiol is an emerging anti-epileptic treatment for refractory epilepsy, a chronic brain network disorder. Refractory epilepsy patients responsive to cannabidiol treatment showed characteristically distinct patterns of brain network activity relative to non-responders. Brain network activity measures may help identify refractory epilepsy patients most likely to benefit from cannabidiol treatment.

## Graphical Abstract

**Graphical Abstract:**
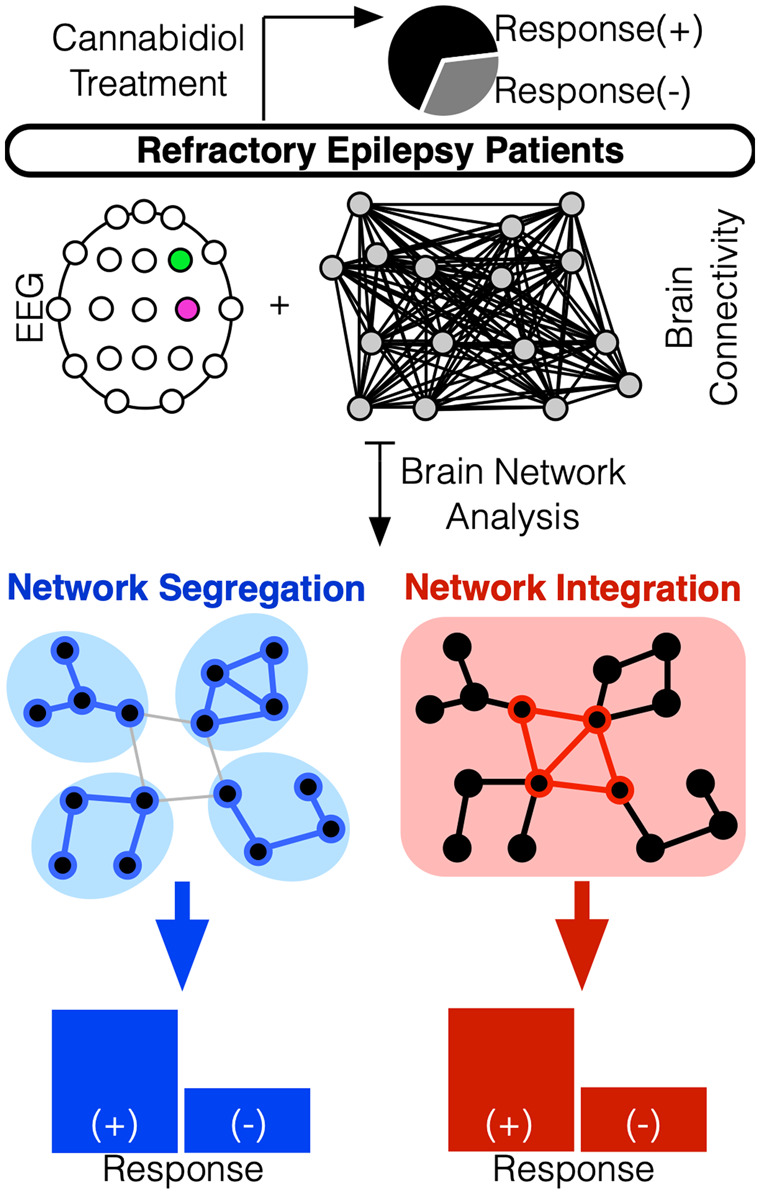

## Introduction

Epilepsy affects ∼1% of the population and is a common cause of morbidity (Zack and Kobau, 2017). Seizures are well controlled by pharmacotherapy alone in ∼70% of patients. Refractory epilepsy, by contrast, affects the remaining 30% of patients and represents an inability to control seizures despite the use of at least two conventional anti-epileptic drugs (AED) at sufficient doses (Kwan and Brodie, 2000). Lennox Gastaut Syndrome (LGS) and Dravet Syndrome (DS) are the two well-recognized refractory epilepsy syndromes and typically present with severe manifestations including multiple seizure types, cognitive impairment, and reduced quality of life. Numerous experimental and clinical approaches have been unsuccessful in sufficiently treating LGS and DS and newer approaches are being explored as well.

Endogenously derived cannabinoids have been a source of interest for treating many neurologic disorders, such as spasticity, pain, nausea and epilepsy (Rosenberg *et al.*, 2015). The pharmacological interest in cannabis compounds arose after the identification of two major neuroactive components—tetrahydrocannabinol and the non-psychotropic cannabidiol (CBD)—and the discovery of an endogenous cannabinoid-signalling pathway (Katona, 2015). Early work has described the potential anti-epileptic properties of cannabinoid-based products (Karler *et al.*, 1973). CBD, the second most abundant phytocannabinoid extracted mainly from cannabis sativa and cannabis indica, has demonstrated anti-seizure activity with a manageable side effect profile (Gloss and Vickrey, 2014). The exact molecular target(s) by which CBD exerts its pharmacological properties are still undetermined (Ibeas Bih *et al.*, 2015). Despite its low affinity for cannabinoid receptors, CBD is able to antagonize cannabinoid type 1 receptor and type 2 receptor agonists *in vitro* with unexpectedly high potency (Thomas *et al.*, 2007), which may explain its lack of psychotropic effects (McPartland *et al.*, 2015). Accordingly, the therapeutic properties of CBD in neurological disorders emerge separately form the endocannabinoid-signalling pathway. CBD is believed to influence neuronal hyperexcitability via several possible mechanisms, including reducing neurotransmitter release (Pazos *et al.*, 2013), activating neurotransmitter receptors (Pazos *et al.*, 2013), and inhibiting neurotransmitter reuptake (Ahrens *et al.*, 2009; Xiong *et al.*, 2012).

There has been particular interest in the use of CBD for treating refractory epilepsy (Devinsky *et al.*, 2014). CBD has shown anticonvulsant activity in many acute animal models of seizures, including temporal lobe epilepsy and partial epilepsy (Jones *et al.*, 2012), though limited data are available in animal models of chronic epilepsy and epileptogenesis (dos Santos *et al.*, 2015). CBD demonstrates variable pharmacokinetic and pharmacodynamic interactions with other AEDs. For example, CBD increases the antiseizure effects of phenytoin and decreases the antiseizure effects of clonazepam and ethosuximide (Consroe and Wolkin, 1977). Drug–drug interactions among AEDs and CBD could be linked to the ability of CBD to inhibit several isoforms of cytochrome P450 (CYP450) resulting in prolonged half-lives of other AEDs and resultant increased therapeutic effectiveness (Zendulka *et al.*, 2016). Recent studies have reported how the drug–drug interaction between CBD and clobazam, which are both catalysed by CYP450 (CYP2C9 and CYP3A4) pathway, could be therapeutically useful in children with refractory epilepsy using such mechanisms (Geffrey *et al.*, 2015).

Recent clinical studies have evaluated the efficacy of CBD in treating refractory epilepsies (Devinsky *et al.*, 2016; Paolino *et al*, 2016). Randomized, placebo-controlled trials using Epidiolex (GWP42003: Greenwich Pharmaceuticals), a highly purified and concentrated CBD medication, have demonstrated clinically significant reduction of seizure frequency in LGS and DS (Devinsky *et al.*, 2017; Thiele *et al.*, 2018). These trials have subsequently led to the US Food and Drug Administration approval of Epidiolex for the treatment of refractory seizures in LGS and DS. Several open-label studies have been conducted through state-sponsored Expanded Access Program mechanisms. Expanded Access Program studies have focused on expanding the understanding of side effects and interactions associated with Epidiolex and exploring its clinical effectiveness in treating multiple seizure types in other epilepsy syndromes as well. Previous studies have primarily relied on self-reported, rather than objective, responses to treatment as documented by patients and families or caretakers (Devinsky *et al.*, 2016). Through our local state-sponsored Expanded Access Program, we sought to perform objectively guided analyses of serially performed electroencephalography (EEG) from our refractory epilepsy patients treated with CBD.

Neurological diseases can be characterized by abnormal patterns of synchronized oscillatory neural activity (Uhlhaas and Singer, 2006; Bowyer, 2016). Cognitive and behavioural functions show clear links with patterns of synchronized oscillatory neural activity (Fries, 2005, 2015). Better understanding the effects of CBD on abnormal patterns of oscillatory neural activity and disease pathophysiology may lead to better quality of life in refractory epilepsy patients by modifying these networks, reducing seizure burden, and possibly improving cognitive function as well. It remains unknown whether CBD alters oscillatory neural activity, though some studies have examined changes in patterns of functional connectivity. Bhattacharyya *et al.* (2015) measured functional magnetic resonance imaging (fMRI) 1 hour after administering either CBD (600 mg) or placebo (flour) on two study visits separated by at least 1 month (Bhattacharyya *et al.*, 2015). Using within-subject contrasts to evaluate CBD-related changes functional connectivity patterns, the authors found that: (i) regions in the striatum showed an increase in connectivity with the caudate nucleus and inferior frontal gyrus, and a decrease in connectivity with the anterior cingulate and medial frontal gyrus; (ii) regions in the inferior frontal gyrus showed a decrease in connectivity with the insula, cerebellum and thalamus; and (3) regions in the hippocampus showed a decrease in connectivity with the anterior cingulate and medal frontal gyrus. These findings suggest CBD may alter patterns of synchronized oscillatory neural activity (Goense *et al.*, 2012). Here, our goal was to use established graph theoretical analyses of EEG phase coherence to better understand how CBD alters oscillatory neural activity resulting in altered cerebral networks and, in turn, how changes in patterns of oscillatory neural activity and network dynamics may predict refractory epilepsy patients most likely to show improvement in seizure outcomes following CBD treatment.

## Materials and methods

### Study design and methods

Patients were enrolled in an open label interventional study starting in 2014. Refractory epilepsy patients with LGS and DS were initially the only eligible diagnosis for this study; during the expanded access phase, patients with other refractory epilepsies were also eligible. Patients remained on their current AEDs, which were adjusted as needed during the study. The non-LGS, non-DS patients were not included in our analyses as they were a heterogeneous group with diverse diagnoses of epilepsies and were small in number (n = 8).

All patients received a 30-minute EEG at the start of inclusion into the study, prior to initiation of CBD therapy. They then received a second 30-minute EEG after being on CBD therapy for 1 month, and a third 30-minute EEG 12 months after their second EEG. All patients thus underwent three routine EEGs at onset, after 1 month, and 13 months into the study.

Patients were treated with Epidiolex, a commercially available version of CBD. All patients were CBD naïve at the time of their first routine EEG with one exception. Patients were started on the product at a dose of 5 mg/kg/day in twice-daily divided doses. Dose escalation was set to 5 mg/kg every 1–2 weeks as tolerated. Patients were monitored via regular office visits every 1-to-3 months. Periodic phone calls were conducted by study personnel to obtain information regarding seizure occurrence or side effects. Patients were instructed to maintain a seizure diary and record any concerning side effects during study procedures.

Therapeutic responsiveness to CBD was evaluated based on changes in the frequency of self-reported seizures. Patients were classified as ‘responders’ if they had greater than a 70% reduction in seizure frequency over the course of the study. Patients were classified as ‘non-responders’ if they had less than a 70% reduction in seizure frequency. Absolute seizure reduction was used as the outcome measure, and we did not study changes in frequencies of different seizure subtypes due to limitations in documentation making it harder to correctly gather this information.

### EEG acquisition and processing

EEG data were recorded using a Natus XLTEC 7.1.1 video-EEG system (Natus, Oakville, ON, Canada) with gold cup electrodes distributed across the scalp according to the International 10-20 System. EEG was measured from 20 scalp electrodes (C3, C4, O1, O2, Cz, F3, F4, F7, F8, Fz, Fp1, Fp2, Fpz, P3, P4, Pz, T3, T4, T5, T6) and two mastoid electrodes (A1, A2), with a ground reference electrode placed at the forehead. Impedances of all electrodes were maintained below 10 kΩ. EEG signals were digitized at a sampling rate of 512 Hz and re-referenced offline to mathematically averaged left and right mastoids (A1–A2). At least 1 h of EEG data were acquired at each study visit. Raw data were visually inspected and epochs containing continuous EEG activity were extracted for further analysis. EEG epochs were 29.0 ± 2.3 minutes in duration.

### EEG phase coherence analysis

#### Band-pass filtering EEG signal

All analyses were performed using MATLAB in conjunction with the Signal Processing and Circular Statistics Toolboxes. Raw EEG signals were band-pass filtered using a two-way least-squares finite impulse response filter (fir1.m function, Signal Processing Toolbox). This filtering method uses a zero-phase forward and reverse operation, which ensures that phase values are not distorted, as can occur with forward-only filtering methods.

#### Extracting instantaneous phase

A Hilbert Transform (hilbert.m function, Signal Processing Toolbox) was then applied to filtered waveforms to extract instantaneous power and phase values. The Hilbert Transform produces the complex analytic signal, $z\left(t\right)$, of the filtered EEG, $f\left(t\right)$, where $z\left(t\right)=\tilde{f}\left(t\right)+i\tilde{f}\left(t\right)=A\left(t\right)e^{i\phi (t)}$, from which instantaneous power, *A(t)*, and instantaneous phase, *iϕ*(*t*), was extracted; $\tilde{f}\left(t\right)$ is the Hilbert Transform of $f\left(t\right)$ and $i=\sqrt{-1}$. Instantaneous phase was estimated using the following equation:

$$\mathrm{Phase}=\arctan\left(\frac{\mathrm{imag}\left(z\left(t\right)\right)}{\mathrm{real}\left(z\left(t\right)\right)}\right).$$

Instantaneous phase assumes values within (−π, π] radians with a cosine phase, such that ± π radians correspond to the troughs and 0 radians to the peak.

#### Estimating phase coherence

Phase coherence, which describes the degree of covariation in oscillatory activity observed between two different channels (Fries, 2005), was estimated across all possible 190 electrode pairs. First, we band-pass filtered the raw EEG signal from channels *k* and *l* according to the following frequency ranges: 1–3 Hz (delta), 4–7 Hz (theta), 8–12 Hz (alpha) and 13–30 Hz (beta). Next, we applied the Hilbert transform to extract instantaneous phase values (in radians, ranging from −π to π). Phase coherence (C) was estimated between electrodes *k* and *l* across each EEG epoch:

$$C_{kl}=\;\left\|\frac{1}{n}\sum_{j=1}^{n}e^{i(\varphi_{j,k}-\varphi_{j,l})}\right\|.$$

This phase coherence measure (circ_r.m function, Circular Statistics Toolbox) varies between 0 (phase independent signals) and 1 (phase dependent signals).

To calculate reliability of phase coherence estimates, EEG epochs were segmented into first and second halves for each electrode. Phase coherence analyses were performed separately on each segment across all 190 possible electrode pairs, resulting in 190 phase coherence estimates for the first segment and 190 phase coherence estimates for the second segment. Next, correlations were calculated between phase coherence estimates for the first and second segments across each electrode pair, and split-half reliability was calculated according to the Spearman–Brown prophesy formula (Brown, 1910; Spearman, 1910). The resulting 190 split-half reliability estimates were then sorted for each frequency band, and quartile analyses were performed to inspect the proportion of electrode pairs showing good (>0.8) reliability.

Topographic maps were constructed to visualize changes in inter-electrode phase coherence. Between- and within-group changes in phase coherence were evaluated using independent- and dependent-sample *t*-tests for each electrode pair. Between-group analyses focused on evaluating how CBD dose (high versus low) and response (responder versus non-responder) influenced CBD-related changes in phase coherence. For visualization purposes, only electrode pairs with significant *t*-values were included in topographic maps.

### Graph theoretical analysis of EEG phase coherence

Our primary goal was to evaluate how global brain network dynamics change with CBD therapy. To this end, we used graph theoretical analyses to describe topographic networks of EEG inter-electrode phase coherence (Bullmore and Sporns, 2009; Rubinov and Sporns, 2010). A graph is a simple model of a network that is described by a set of nodes (i.e. electrodes) and edges (i.e. phase coherence values) that characterize connections between each node. Here, we used graph theoretic analyses to estimate undirected complex network measures using a combination of EEGNET (Hassan *et al.*, 2015) and Brain Connectivity Toolbox (Rubinov and Sporns, 2010). Given our goal of evaluating global changes in brain network synchronization, we focused on graph theoretic metrics of function segregation and integration. *Functional integration* corresponds to the ease to which information can be rapidly communicated across distributed brain regions. Measures of functional integration are characterized based on path length within the network, where a path is a sequence of consecutive edges in a graph and its length is the number of edges traversed between two nodes. *Functional segregation* corresponds to the degree to which distributed brain regions can be partitioned into highly interconnected groups of separable nodes. Measures of functional segregation are characterized by the presence of modular structure within the network. Specifically, a triplet is defined as three nodes connected by either two (i.e. open triplet) or three (i.e. closed triplet) edges. Closed triplets (i.e. triangles) are representative of more modular, and therefore segregated, structures.

Briefly, inter-electrode phase coherence values were sorted into an *N* × *N* matrix comprised of *L* cells, where *N* is the number of EEG electrodes (or nodes) and *L* is the number of inter-electrode connections (or edges). Phase coherence matrices were thresholded to include the 30% of edges with the highest phase coherence values, resulting in a graph matrix composed of *n* nodes and *l* edges. From this matrix, we obtained the following global undirected network measures, using Brain Connectivity Toolbox (Rubinov and Sporns, 2010) terminology and definitions to describe them here.

*Degree* is defined as the number of nodes connected to any given node through existing edges within the graph. For each node *i*, degree *k* was calculated as:

$$k_{i}=\sum_{j\in N}a_{ij},$$

where (*i, j*) is an edge between nodes *i* and *j, N* is the set of all nodes in the network, and *a_ij_* is the connection status between nodes *i* and *j*, where *a_ij_* = 1 when edge (*i, j*) exists and *a_ij_* = 0 when edge (*i, j*) does not exist within the graph. Focusing on global brain network dynamic metrics, we extracted maximum degree across all nodes. Larger degree values are associated with networks having a larger number of edges linked to a single node.

*Characteristic path length* is defined as the average shortest path length between any two nodes *i* and *j* among all available edges within the graph. Characteristic path length *L* was calculated as (Watts and Strogatz, 1998):

$$L=\;\frac{1}{n}\sum_{i\in N}L_{i}=\;\frac{1}{n}\sum_{i\in N}\frac{\sum_{j\in N\;j\neq i}d_{ij}}{n-1},$$

where *L_i_* is the average distance between node *i* and all other nodes, and *d_ij_* is the shortest path length between nodes *i* and *j*, calculated as:

$$d_{ij}=\;\sum_{a_{uv}\in gi↔j}a_{uv},$$

where gi↔j is the shortest geodesic path between nodes *i* and *j*. Larger characteristic path length values are associated with poor network integration as more edges are needed to connect two given nodes.

*Global efficiency* is defined as the average inverse shortest path length between any two nodes *i* and *j* among all available edges within the graph. Global efficiency *E* was calculated as (Latora and Marchiori, 2001):

$$E=\;\frac{1}{n}\sum_{i\in N}E_{i}=\;\frac{1}{n}\sum_{i\in N}\frac{\sum_{j\in N\;j\neq i}d_{ij}^{-1}}{n-1},$$

where *E_i_* is the efficiency of node *i*. Larger global efficiency values are associated with better network integration as fewer edges are needed to connect two given nodes.

*Modularity* is defined as the degree to which the network can be subdivided into non-overlapping modules of nodes that maximize the number of within-module edges and minimizes the number of between-module edges. Modularity *Q* is calculated as (Newman, 2006):

$$Q=\;\frac{1}{l}\sum_{i,j\in N}\left(a_{ij}-\frac{k_{i}k_{j}}{l}\right)\delta_{m_{i},m_{j}},$$

where *m_i_* is the module containing node *i*, and $\delta_{m_{i},m_{j}}$ = 1 if *m_i_* = *m_j_*, and 0 when *m_i_* ≠  *m_j_*. Larger modularity values are associated with greater network segregation and complexity due to highly segregated clusters without many connections between them.

*Transitivity* is defined as the ratio of triangles to triplets in the network, corresponding to the fraction of node’s neighbours that are neighbours of each other. Transitivity *T* is calculated as (Newman, 2003):

$$T=\;\frac{\sum_{i\in N}{2t}_{i}}{\sum_{i\in N}k_{i}(k_{i}-1)},$$

where *t_i_* is the number of triangles around a node *i*, calculated as:

$$t_{i}=\;\frac{1}{2}\sum_{j,h\in N}a_{ij}a_{ih}a_{jh}.$$

Larger transitivity values are associated with weaker network segregation due to stronger overlapping neighbourhood structure.

To summarize, we measured network degree, characteristic path length, global efficiency, modularity and transitivity. Characteristic path length and global efficiency are measures of network integration, where greater network integration is associated with smaller characteristic path length and larger global efficiency values. Modularity and transitivity are measures of network segregation, where greater network segregation is associated with larger modularity and smaller transitivity values.

### Statistical analysis

Patient data were individually summarized (Table 1). Categorical data (gender, previous vagal nerve stimulation and CBD response) were descriptively summarized using frequency and percentage tables. Numeric data (age, number of AEDs and CBD dosage) were descriptively summarized using means and standard deviations. Correlations were used to assess the relationship between age, number of AEDs and CBD dosage. Patients were divided into those who either showed positive (‘responders’) or no response (‘non-responders’) to CBD treatment, and independent-sample *t*-tests were performed to evaluate group differences on age, number of AEDs and CBD dosage, and chi-squared tests were performed to evaluate group differences on gender. Generalized linear modelling procedures were used to perform logistic regression to determine whether CBD response was associated with age, gender, number of AEDs and CBD dosage.

**Table 1:** Patient characteristics

ID	Age (years)	Gender (M/F)	Pathology/prior surgeries	AEDs (#)	Vagal nerve stimulation (Y/N)	Other treatments	Dose (mg/kg)	Response (Y/N)
1	16	M	Callosotomy+R post+Lmesial resec+multi transections	3	Y	None	25	N
2	32	F	l hemi atro, L schizen, dysplasia, Polymicro/pachygyria	4	N	None	25	Y
3	12	F	Ant corpus callosotomy+	4	Y	None	5	Y
4	25	M	Callosotomy+mild diff atrophy	4	Y	None	25	N
5	24	M	Callosotomy + L fron lobec + R fron small resec	3	Y	None	5	N
6	15	M	R fron lobec + callosotomy	3	Y	None	25	N
7	20	M	Cerebellar hyperintensity − type?	3	Y	None	15	Y
8	27	M	Lissencephaly	4	Y	None	20	Y
9	17	M	Callosotomy+	4	Y	None	20	Y
10	22	F	Mild diffuse atrophy	3	Y	None	15	N
11	12	M	None	3	Y	None	20	Y
12	36	M	Non-lesional	5	N	None	30	Y
13	7	F	None	2	N	None	15	Y
14	15	M	None	4	Y	None	15	Y
15	10	M	Septo optic dyspl, b/l heterotopia, septal dysgenesis	4	Y	None	20	Y

Between-group differences (high versus low CBD dose; responder versus non-responder) and within-group changes in repeated measures were assessed using linear mixed models in SAS software version 9.4 (SAS Institute Inc., Cary, NC, USA). Repeated measures were modelled using a compound symmetry covariance structure. Kenward–Roger degrees of freedom corrections were used to account for missing data (Kenward and Roger, 1997). Omnibus statistics was evaluated for effects of group and visit, and group-by-visit interactions. Effects of age on graph theoretical outcome measures were evaluated to determine whether age should be included as a covariate in linear mixed models. *Post hoc* within- and between-group contrasts were assessed by comparing model-derived least square means. Statistical significance was set to the standard *P* < 0.05 level. We also estimated standardized effect sizes (Cohen’s d) (Rosnow and Rosenthal, 1996) for main effects and reported those that exceeded a medium effect size of 0.5 in cases in which effects were trending.

### Data availability

Data and analysis scripts supporting the findings of this study are available from the corresponding author, upon reasonable request.

## Results

LGS and DS patients (*n* = 15) enrolled into an open label CBD study. Patients were started on CBD at a dose of 5 mg/kg/day in twice daily divided doses, and dose escalation was set to 5 mg/kg every 1–2 weeks as tolerated. Patients were monitored via regular office visits every 1–3 months to obtain information regarding seizure occurrence and side effects. Therapeutic responsiveness was evaluated based on changes in the frequency of self-reported seizures. Patients were classified as ‘responders’ (or ‘non-responders’) if they had greater than (or less than) 70% reduction in seizure frequency by the end of the study.

All patients underwent 30-minute EEGs prior to initiating CBD treatment (T1), after 1-month of CBD treatment (T2) and after 13 months of CBD treatment (T3). EEG phase coherence was estimated as follows (see Materials and methods for further information). First, raw EEG signals for all 20 scalp electrodes were band-pass filtered into delta (1–3 Hz), theta (4–7 Hz), alpha (8–12 Hz) and beta (13–30 Hz) frequency bands. Filtered signals then underwent Hilbert transform to extract instantaneous phase values. EEG phase coherence was then estimated by calculating the phase locking value (Lachaux *et al*., 1999) for all possible 190 electrode pairs.

### Clinical characteristics

 Patients were 19.3 ± 8.3 years of age (range: 7–36) and 73% male (11/15 patients) (Table 1). Patients were being treated with 3.5 ± 0.7 AEDs and 53% (8/15) of patients were being treated with more than three AEDs. Eighty per cent of patients (12/15 patients) had previously undergone vagal nerve stimulation. CBD dosage was ∼18.7 ± 7.2 mg/kg, where 60% of patients (9/15) were treated with CBD dosages greater than 15 mg/kg and 33.3% of patients (5/15) were treated with CBD dosages greater than 20 mg/kg. Age was associated with number of AEDs (*r* = 0.56, *P* = 0.03). We were unable to detect an association between age and CBD dosage (*r* = 0.39, *P* = 0.15), and number of AEDs and CBD dosage (*r* = 0.34, *P* = 0.21).

In the current trial, 10 of the 15 enrolled patients (67%) responded positively to CBD treatment and showed a significant reduction in seizure frequency (>70% seizure reduction). Of the 10 responders, 6 were seizure free while the remaining 4 had 70–95% seizure reduction on CBD therapy. Patients who responded positively were indistinguishable from patients showing no response with respect to age (*t*_(13)_ = 0.34, *P* = 0.74), gender (χ^2^_(1)_ = 0.17, *P* = 0.68), number of AEDs (*t*_(13)_ = −1.25, *P* = 0.23) and CBD dosage (*t*_(13)_ = 0.12, *P* = 0.90). Similarly, logistic regression procedures failed to detect an effect of age (χ^2^_(1)_ = 1.72, *P* = 0.19), gender (χ^2^_(1)_ = 1.83, *P* = 0.18), number of AEDs (χ^2^_(1)_ = 1.98, *P* = 0.16) and CBD dosage (χ^2^_(1)_ = 1.02, *P* = 0.31) on CBD response.

Study patients generally responded positively to the CBD trial, leading to a reduction in seizure frequency. Clinical variables were unable to predict which patients would respond positively. Developing objective predictors of therapeutic responsiveness to CBD would greatly improve treatment decisions and outcomes. To this end, we evaluated how CBD dosage and therapeutic outcomes are related to putative changes in global patterns of brain synchronization and brain network dynamics across each frequency band (Table 2).

**Table 2:** Graph theoretical outcome measures for each frequency band and study visit

Frequency band	Graph theoretical measure	Visit 1 (*n* = 15)	Visit 2 (*n* = 15)	Visit 3 (*n* = 13)
Delta	Degree	13.40 ± 1.55	14.47 ± 2.42	14.23 ± 1.96
	Efficiency	0.74 ± 0.04	0.76 ± 0.08	0.75 ± 0.06
	Modularity	0.22 ± 0.05	0.19 ± 0.09	0.21 ± 0.06
	Transitivity	0.42 ± 0.06	0.46 ± 0.11	0.44 ± 0.10
Theta	Degree	9.13 ± 2.20	9.73 ± 3.28	9.31 ± 2.53
	Efficiency	0.53 ± 0.11	0.56 ± 0.11	0.55 ± 0.11
	Modularity	0.30 ± 0.11	0.28 ± 0.13	0.28 ± 0.13
	Transitivity	0.49 ± 0.10	0.48 ± 0.13	0.48 ± 0.13
Alpha	Degree	6.33 ± 2.26	6.40 ± 2.82	5.69 ± 1.49
	Efficiency	0.33 ± 0.10	0.31 ± 0.13	0.33 ± 0.09
	Modularity	0.40 ± 0.12	0.40 ± 0.17	0.46 ± 0.11
	Transitivity	0.43 ± 0.15	0.43 ± 0.18	0.41 ± 0.12
Beta	Degree	4.33 ± 1.59	4.47 ± 1.81	4.85 ± 2.03
	Efficiency	0.23 ± 0.12	0.21 ± 0.13	0.25 ± 0.13
	Modularity	0.50 ± 0.13	0.44 ± 0.133	0.51 ± 0.15
	Transitivity	0.32 ± 0.17	0.29 ± 0.17	0.35 ± 0.15

### Reliability of phase coherence estimates

**Figure 1:**
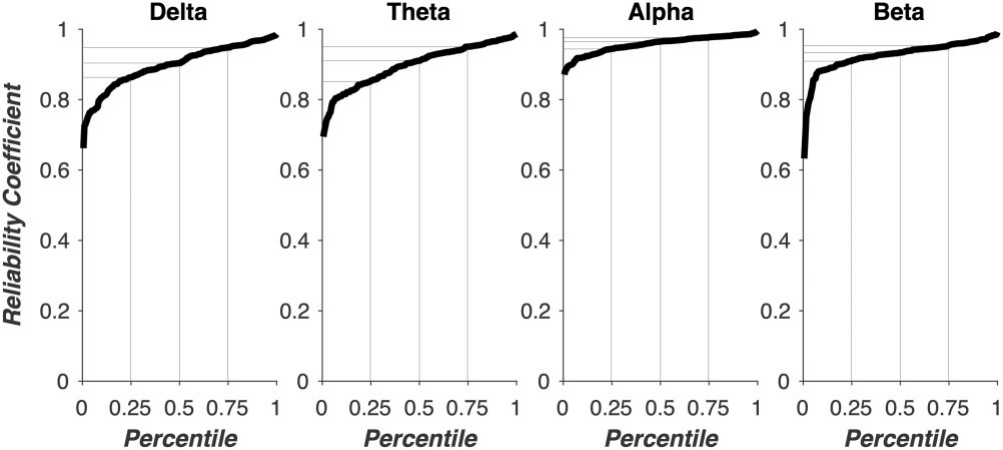
**Reliability of phase coherence estimates.** Each subplot shows split-half reliability coefficients for each frequency band. EEG recordings were divided into two equal segments and phase coherence analyses were performed separately on each half. Split-half reliability was then estimated on phase coherence estimates from each segment using the Spearman–Brown prophesy formula. Percentiles are marked in grey lines. Reliability for the delta frequency band (1–3 Hz) was greater than 0.86, 0.90 and 0.94 for the 25th, 50th and 75th quartile, respectively. Reliability for the theta frequency band (4–7 Hz) was greater than 0.85, 0.91 and 0.95 for the 25th, 50th and 75th quartile, respectively. Reliability for the alpha frequency band (8–12 Hz) was greater than 0.94, 0.96 and 0.97 for the 25th, 50th and 75th quartile, respectively. Reliability for the beta frequency band (13–30 Hz) was greater than 0.90, 0.93 and 0.95 for the 25th, 50th and 75th quartile, respectively.

We first evaluated the split-half reliability of phase coherence estimates at T1 before assessing CBD treatment-related changes (Fig. 1). Reliability was above 0.6 for all 760 (190 electrode pairs * 4 frequency bands) phase coherence estimates. Reliability for the delta frequency band (1–3 Hz) was greater than 0.86, 0.90 and 0.94 for the 25th, 50th and 75th quartile, respectively. Reliability for the theta frequency band (4–7 Hz) was greater than 0.85, 0.91 and 0.95 for the 25th, 50th and 75th quartile, respectively. Reliability for the alpha frequency band (8–12 Hz) was greater than 0.94, 0.96 and 0.97 for the 25th, 50th and 75th quartile, respectively. Reliability for the beta frequency band (13–30 Hz) was greater than 0.90, 0.93 and 0.95 for the 25th, 50th and 75th quartile, respectively. These results demonstrate good reliability for phase coherence estimates across each electrode pair and frequency band and suggest we can be confident in interpreting changes in phase coherence estimates.

### Effects of age on global brain network dynamics

We evaluated effects of age on graph theoretical measures of global brain network dynamics derived from topographic patterns of EEG phase coherence across each frequency band. In the delta frequency band, effects of age were observed in path length [*F*(1,10.2) = 5.52, *P* = 0.040; Cohen’s *d* = 1.40; ß = 0.0089 ± 0.0038], degree [*F*(1,10.6) = 5.87, *P* = 0.035; Cohen’s *d* = 1.42; ß = 0.0386 ± 0.0159] and transitivity [*F*(1,12.1) = 14.85, *P* = 0.0023; Cohen’s *d* = 2.13; ß = 0.0067 ± 0.0017]. In the theta frequency band, effects of age were observed in global efficiency [*F*(1,12.1) = 11.46, *P* = 0.0054; Cohen’s *d* = 1.87; ß = −0.0032 ± 0.00095], degree [*F*(1,13) = 6.60, *P* = 0.0234; Cohen’s *d* = 1.37; ß = 0.0488 ± 0.019], modularity [*F*(1,12.7) = 6.18, *P* = 0.0277; Cohen’s *d* = 1.34; ß = −0.004 ± 0.0016] and transitivity [*F*(1,12.8) = 15.85, *P* = 0.0016; Cohen’s *d* = 2.14; ß = 0.00699 ± 0.0018]. Effects of age were observed in transitivity within alpha [*F*(1,12.1) = 6.58, *P* = 0.0247; Cohen’s *d* = 1.42; ß = 0.00398 ± 0.00155] and beta [*F*(1,12.8) = 4.34, *P* = 0.0578; Cohen’s *d* = 1.12; ß = 0.0026 ± 0.0012] frequency bands. Given the widespread effects of age on global brain network dynamics, we included age as a covariate in subsequent modelling procedures.

### CBD dosage and changes in global brain network dynamics

**Figure 2:**
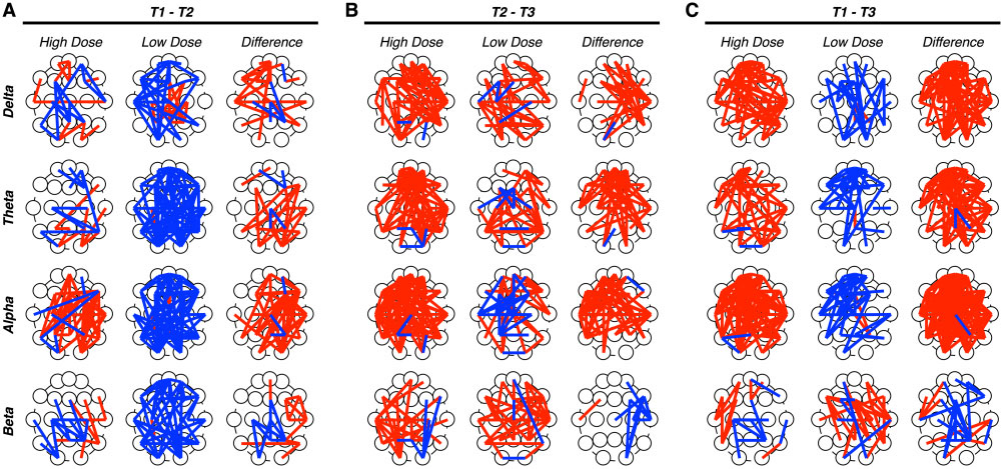
**Evaluating effects of treatment dosage on CBD-related changes in phase coherence.** Topographic maps of electrode pairs showing an increase (red lines) or decrease (blue line) in phase coherence for each frequency band between T1 and T2 (**A**), T2 and T3 (**B**) and T1 and T3 (**C**) measurements. Patients were separated into high (left) and low (middle) CBD dosage groups; topographic maps of between-group differences were constructed (right). (**A**) Between T1 and T2, relatively greater reductions in phase coherence were observed in the low dosage relative to the high dosage groups across all frequency bands. (**B**) Between T2 and T3, relatively greater increases in phase coherence were observed in the high dosage relative to the low dosage groups in the delta, theta and alpha frequency bands. (**C**) Between T1 and T3, relatively greater increases in phase coherence were observed in the high dose relative to the low dose group in the delta, theta and alpha frequency bands.

To evaluate the association between CBD dosage and changes in global brain network dynamics, we first separated patients into high and low dosage groups using a median split on CBD dosage (Fig. 2). Comparing topographic patterns of phase coherence between T1 and T2 measurements (Fig. 2A) revealed relatively greater reductions in phase coherence in the low dosage relative to the high dosage groups across all frequency bands. Comparing topographic patterns of phase coherence between T2 and T3 measurements (Fig. 2B) revealed relatively greater increases in phase coherence in the high dosage relative to the low dosage groups in the delta, theta, and alpha frequency bands. Comparing topographic patterns of phase coherence between T1 and T3 measurements (Fig. 2C) similarly revealed greater increases in phase coherence in the high dosage relative to the low dosage groups in the delta, theta and alpha frequency bands, and greater decreases in phase coherence in the high dosage relative to the low dosage groups in the beta frequency band. Together, these results suggest high CBD dosage is associated with greater CBD-related increases global phase coherence.

Next, we evaluated effects of dose and visit, as well as dose-by-visit interaction, obtained from linear mixed modelling procedures on graph theoretical outcome measures. Dose was included as a continuous variable in modelling procedures. In the delta frequency band, we observed a significant effect of dose in global efficiency [*F*(1,12.1) = 10.63, *P* = 0.0068; Cohen’s *d* = 1.92; ß = 0.0029 ± 0.002] and transitivity [*F*(1,12.3) = 9.40, *P* = 0.0096; Cohen’s *d* = 1.68; ß = −0.0054 ± 0.002]. In the theta frequency band, we similarly observed a significant effect of dose in global efficiency [*F*(1,13.5) = 7.42, *P* = 0.0169; Cohen’s *d* = 1.43; ß = 0.0052 ± 0.0016] and transitivity [*F*(1,13.6) = 20.03, *P *= 0.0006; Cohen’s *d* = 2.34; ß = −0.0091 ± 0.0012]. In the alpha frequency band, global efficiency showed effects of dose [*F*(1,11.5) = 5.26, *P* = 0.042; Cohen’s *d* = 1.30; ß = 0.0038 ± 0.0015], visit [*F*(2,25.3) = 2.88, *P* = 0.075; Cohen’s *d* = 0.65], and a dose-by-visit interaction [*F*(2,24.8) = 2.92, *P* = 0.073; Cohen’s *d* = 0.66]. Trending effect of visit was driven by increase in global efficiency at visit 3 [*t*(27) = 1.86, *P* = 0.074; ß = 0.090 ± 0.048], but not visit 2 (*P* = 0.57), relative to visit 1. The trending interaction was suggestive of a significant decrease in the global efficiency by CBD dose slop at visit 3 [*t*(26.1) = −2.07, *P* = 0.049; ß = −0.0049 ± 0.0023], but not visit 2 (*P* = 0.85), relative to visit 1. In addition, transitivity showed a trending effect of dose [*F*(2,25.3) = 2.88, *P* = 0.075; Cohen’s *d* = 0.65; ß = −0.0038 ± 0.0015] in the alpha frequency band.

### Therapeutic responsiveness and changes in global brain network dynamics

**Figure 3:**
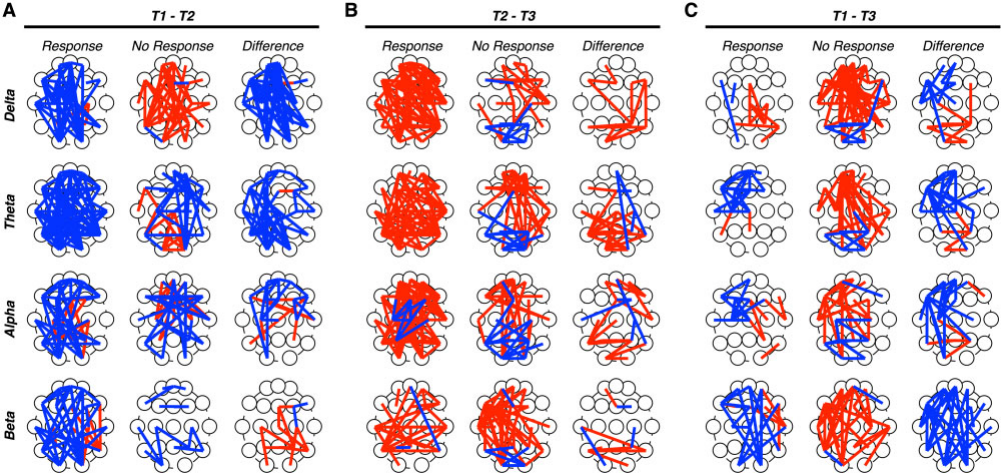
**Evaluating differences in CBD-related changes in phase coherence between responders and non-responders.** Topographic maps of electrode pairs showing an increase (red lines) or decrease (blue line) in phase coherence for each frequency band between T1 and T2 (**A**), T2 and T3 (**B**) and T1 and T3 **(C)** measurements. Patients were separated into responder (left) and non-responder (middle) groups; topographic maps of between-group differences were constructed (right). (**A**) Between T1 and T2, relatively greater reductions in phase coherence were observed across all frequency bands in the responder relative non-responder group. (**B**) Between T2 and T3, relative greater increases in phase coherence observed across all frequency bands in both groups. (**C**) Between T1 and T3, relative greater increases in phase coherence were observed in the non-responder group, whereas the responder group showed greater reductions in phase coherence.

To evaluate the association between global brain network dynamics and therapeutic responsiveness, we compared CBD-related changes in global phase coherence across patients classified as either treatment responders or non-responders (Fig. 3). Comparing topographic patterns of phase coherence between T1 and T2 measurements (Fig. 3A) revealed large-scale reductions in phase coherence across all frequency bands in the responder relative to the non-responder group. Comparing topographic patterns of phase coherence between T2 and T3 measurements (Fig. 3B) revealed large-scale increases in phase coherence across all frequency bands in both groups. Comparing topographic patterns of phase coherence between T1 and T3 measurements (Fig. 3C) revealed large-scale increases in phase coherence in the non-responder group, whereas the responder group showed greater reductions in phase coherence. Together, these results indicate that responders, relative to non-responders, showed greater reductions in phase coherence across all frequency bands following CBD treatment.

**Figure 4:**
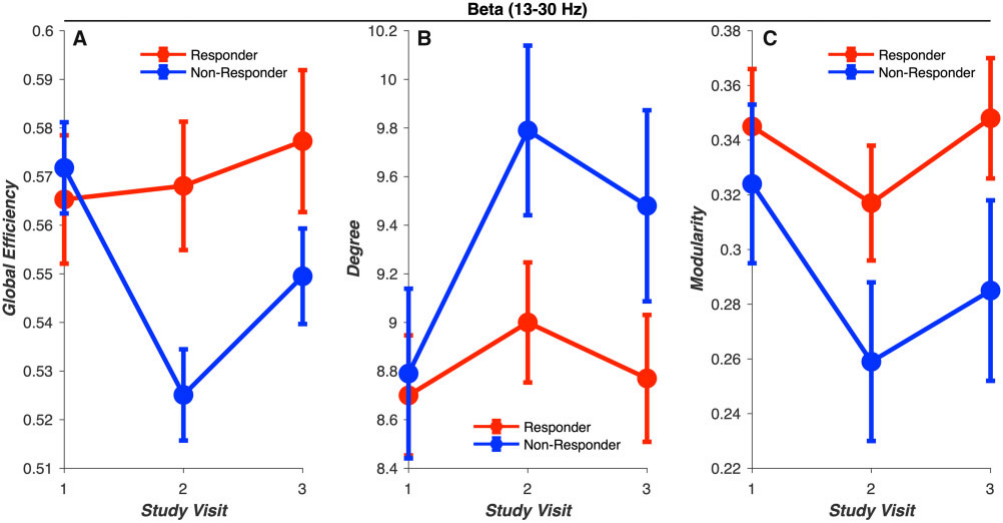
**Comparing graph theoretical measures of global brain network dynamics in CBD responder and non-responder groups.** Between-group differences in graph theoretical measures of global brain network dynamics were observed only in the beta frequency band. (**A**) Global efficiency, a measure of network integration, showed a trending group-by-visit interaction (*P* = 0.074; Cohen’s *d* = 0.74). Higher global efficiency was observed in the responder relative to non-responder group (*P* = 0.013). Greater reductions in global efficiency were observed between visits 1 and 2 in the non-responder (*P* = 0.005) but not responder group (*P* = 0.80). (**B**) Degree, a measure of network connectivity, was lower in the responder relative to non-responder group (*P* = 0.030; Cohen’s *d* = 1.33). (**C**) Modularity, a measure of network segregation, showed a trending effect of group (*P* = 0.093; Cohen’s *d* = 1.01), where higher modularity was observed in the responder relative to non-responder group.

Next, we evaluated effects of group (responder versus non-responder) and visit, as well as group-by-visit interaction, obtained from linear mixed modelling procedures on graph theoretical outcome measures (Fig. 4). In the beta-frequency band, we observed a significant group-by-visit interaction on global efficiency [*F*(2,24.9) = 3.71, *P* = 0.039; Cohen’s *d* = 0.74; Fig. 4A]. *Post hoc* analyses revealed significantly higher global efficiency in the responder group relative to non-responder group in visit 2 assessments [*t*(28.4) = −2.64, *P* = 0.013]. Between-group differences in visit 2 assessments were driven by significant reductions in global efficiency between visit 1 and visit 2 in the non-responder group [*t*(24.4) = −3.08, *P* = 0.005], but not the responder group (*P* = 0.80), though a significant reduction in global efficiency was observed across groups [*t*(24.4) = −2.37, *P* = 0.026]. Furthermore, we observed an effect of group on degree [*F*(1,12.5) = 6.0, *P* = 0.030; Cohen’s *d* = 1.33; Fig. 4B], where degree was lower in the responder relative to non-responder group, and a trending effect of group on modularity [*F*(1,12) = 3.33, *P* = 0.093; Cohen’s *d* = 1.01; Fig. 4C], where modularity was higher in the responder relative to non-responder group, in the beta-frequency band. No effects of treatment responsivity group on graph theoretical measures were observed in the delta, theta and alpha frequency bands.

## Discussion

In this study, we aimed to develop objective measures of responsiveness to CBD treatment for refractory epilepsy by measuring changes in global patterns of brain network dynamics using a combination of EEG phase coherence and graph theoretical methods. We found that patients showing positive responsiveness to CBD treatment showed stronger network integration and segregation exclusively in the beta-frequency band, as demonstrated by larger global efficiency, higher modularity, and lower degree in the responder relative to non-responder group. These results suggest that beta networks in the responder relative to non-responder group were more likely to show: (i) reduced average shortest path length between any nodes, reflecting stronger network integration; (ii) networks that maximize within-module edges and minimize between-module edges, reflecting stronger network segregation and (iii) fewer edges associated with a given node. We also found that larger CBD dosages were associated with stronger network integration and segregation in the delta, theta and alpha frequency bands. Specifically, larger CBD dosages were associated with an increase in global efficiency and a decrease in transitivity. These results suggest brain networks in the 1–12 Hz frequency range (delta, theta and alpha bands) were more likely to show the following in patients receiving relatively higher CBD doses: (i) reduced average shortest path length between any nodes, reflecting stronger network integration and (ii) reduced overlapping neighbourhood structure among nodes, reflecting stronger network segregation. Importantly, we failed to observe a relationship between CBD dosage and responsiveness to treatment, suggesting that CBD responsiveness and dosage were dissociable predictors of changes in global patterns of neural synchronization. Together, these results provide novel insights into how CBD alters global patterns of neural synchronization, and, in turn, how global patterns of neural synchronization may be used to predict those refractory epilepsy patients who are most likely to benefit from CBD even before initiating treatment.

Previous studies have evaluated changes in graph theoretical measures of brain network dynamics in epilepsy patients (Chiang and Haneef, 2014; Haneef and Chiang, 2014). In one study (Horstmann *et al.*, 2010), patients showed an increase in characteristic path length across nearly all frequency bands, excluding alpha, suggesting a broadband reduction in brain network integration. In a separate study (Quraan *et al.*, 2013), by contrast, patients showed an increase in small worldness, characterized by shorter path lengths, in the theta frequency band, and a decrease in small worldness in the alpha frequency band. These conflicting findings highlight our limited understanding of brain network dynamics in epilepsy (Pegg *et al.*, 2020). In the current work, we found that patients showing positive responsiveness to CBD treatment showed stronger network integration (i.e. shorter path lengths) and stronger network segregation (i.e. increased modularity). These findings suggest either: (i) a positive response to CBD treatment is characterized by an increase in network integration and segregation or (ii) patients most likely to respond to CBD treatment show stronger network integration and segregation. Future large-scale confirmatory studies will be critical for disentangling these two alternative explanations. Nevertheless, these results reflect the importance of global brain network dynamics in understanding the mechanisms of CBD as a treatment for refractory epilepsy.

Oscillatory neural activity is mediated by gamma-aminobutyric acid (GABA) ergic interneurons (Gonzalez-Burgos and Lewis, 2008) and cannabinoid type 1 receptor modulate gamma-aminobutyric acid release (Skosnik *et al.*, 2012; Sales-Carbonell *et al.*, 2013). The cannabinoid system participates in the fine-tuning of oscillatory neural activity by regulating the release of gamma-aminobutyric acid and glutamate in an activity-dependent manner (Freund *et al.*, 2003). Cannabinoid type 1 receptor binding and activation of the cannabinoid system may therefore lead to perturbations in oscillatory neural activity and subsequent changes in global neural synchronization (Cortes-Briones *et al.*, 2016). Here, we demonstrate CBD-related changes in patterns of global brain network dynamics characterized by increased functional network integration and segregation. It remains to be determined how differential binding of CBD to cannabinoid type 1 receptor and cannabinoid type 2 receptor produce changes in brain networks dynamics reported here.

Clinical trials frequently evaluate experimental AEDs using self-reported outcome measures of seizure frequency and treatment response. Self-reported outcome measures are subjective by nature and demand patients have sufficient self-awareness to evaluate their symptoms. As a well-proven fact, self-reported seizure frequency is often under- or over-reported (Blum *et al.*, 1996; Blachut *et al.*, 2017). Here, we used EEG phase coherence and graph theoretical analyses to develop objective global brain network dynamic measures of therapeutic responsiveness to CBD. We found that graph theoretical measures of global brain network dynamics were associated with changes in seizure frequency, where strong network integration and segregation were observed in treatment responders relative to non-responders. These findings are consistent with the certainty that epilepsy is a brain network disorder, rather than an isolated cerebral dysfunction (Kramer and Cash, 2012; Kanner *et al.*, 2017). Implementing graph theoretical analysis of EEG phase coherence in refractory epilepsy patients planning to undergo CBD treatment may prove to be a valuable surrogate for self-reported measures of seizure frequency. Furthermore, assessing changes in global brain network dynamics may prove useful for tracking treatment responsiveness and identifying patients who may benefit most from treatment.

Clinical trials evaluate the efficacy of AEDs by setting a therapeutic target of greater than 50% seizure reduction rate with the use of trial medications. Multiple clinical trials have consistently shown that patients receiving these medications, including AEDs, demonstrate a placebo response rate of up to 30%. We found that our responders had greater than 70% seizure reduction with CBD use while the non-responders usually showed minimal or no response. In one exception, we found a single non-responder had a 30–40% reduction in seizures, which was indistinguishable from a placebo response. Consequently, we classified this patient as a non-responder. Nevertheless, CBD treatment yielded a responsivity rate higher than clinical standards.

There are some limitations to our study. First, our limited sample size (*n* = 15) does offer the achievement of statistical significance, or medium-to-high effect sizes in trending effects and the ability to compare two well-matched groups of responders and non-responders. We do accept that more robust multivariate analyses would be limited in power by the sample sizes in our study. Second, most of our patients are in their late teens and early-mid-twenties, rather than most LGS- or DS-based studies, which usually included younger patients below the ages of 10 years. Third, prior treatments may have also affected our results. Vagal nerve stimulation can be eliminated from this consideration because it was used significantly across groups (7/10 responders and 4/5 non-responders). Corpus callosotomy affect brain network dynamics as well, due to its disconnection of both hemispheres and its resultant changes in network synchronization. We posit that the effect of the callosotomy would be minimal, if any, as they had these surgeries many years ago and its resultant effects on network dynamics would be minimal. Third, study duration could be a confounder as we studied our patients for slightly over a year. However, most studies on AEDs usually last for 3–12 months, which we believe would be similarly sufficient for our CBD study to evaluate responsiveness and overcome any placebo or short-term effects. Fourth, the relationship between brain network dynamics and seizure burden in epilepsy has not been clearly established, making it challenging to study efficacy of AEDs on changes in these measures. Finally, we measured absolute seizure reduction in our patients as the major study outcome in our patients. Prior studies have also analysed reduction in seizure subtypes in LGS or DS patients, as some patients may show a significant reduction in disabling seizure types like convulsions or atonic drop attacks rather than overall seizure burden, which would still reinforce the notion of a successful outcome from therapy in these patients, due to improved quality of life from reduction in disabling seizures. We were unable to clearly obtain data from the patient charts regarding reductions in individual seizure subtypes due to limitations in documentation, which forced us to use absolute seizure reduction as the primary outcome measure. Nevertheless, despite the aforementioned limitations, we find the results from our exploratory investigation into measuring the association between brain network dynamics and CBD therapy in refractory epilepsy patients extremely promising and believe that these novel results warrant further and deeper investigation in future studies using larger patient samples and more advanced investigative methods.

In conclusion, we demonstrated global brain network dynamics are associated with therapeutic responsiveness to CBD treatment. These results provide a proof-of-concept demonstration that may be used to better understand how CBD may reduce seizure burden in refractory epilepsy patients and alter their brain network dynamics, which could open up newer avenues for scientific inquiry into the effects of CBD on cognition and behavioural neuroscience at large.

## Funding

This study was supported by the Department of Ophthalmology & Visual Sciences and the Department of Neurological Sciences at the University of Nebraska Medical Center.

## Competing Interests

D.M. serves on the speaker’s bureau for Greenwhich Pharma. D.E.A. and A.S. report no competing interests.

## Abbreviations

**AED** =: anti-epileptic drugs
**BD** =: cannabidiol
**DS** =: Dravet syndrome
**EEG** =: electroencephalography
**fMRI** =: functional magnetic resonance imaging
**LGS** =: Lennox Gastaut syndrome
